# In Vivo Targeted Magnetic Resonance Imaging of Endogenous Neural Stem Cells in the Adult Rodent Brain

**DOI:** 10.1155/2015/131054

**Published:** 2015-10-25

**Authors:** Xiao-Mei Zhong, Fang Zhang, Ming Yang, Xue-Hua Wen, Xiang Zhang, Xiao-Hui Duan, Jun Shen

**Affiliations:** ^1^Department of Radiology, Sun Yat-Sen Memorial Hospital, Sun Yat-Sen University, No. 107 Yanjiang Road West, Guangzhou, Guangdong 510120, China; ^2^Department of Radiology, Zhongda Hospital of Southeast University, Nanjing 210009, China

## Abstract

Neural stem cells in the adult mammalian brain have a significant level of neurogenesis plasticity. In vivo monitoring of adult endogenous NSCs would be of great benefit to the understanding of the neurogenesis plasticity under normal and pathological conditions. Here we show the feasibility of in vivo targeted MR imaging of endogenous NSCs in adult mouse brain by intraventricular delivery of monoclonal anti-CD15 antibody conjugated superparamagnetic iron oxide nanoparticles. After intraventricular administration of these nanoparticles, the subpopulation of NSCs in the anterior subventricular zone and the beginning of the rostral migratory stream could be in situ labeled and were in vivo visualized with 7.0-T MR imaging during a period from 1 day to 7 days after the injection. Histology confirmed that the injected targeted nanoparticles were specifically bound to CD15 positive cells and their surrounding extracellular matrix. Our results suggest that in vivo targeted MR imaging of endogenous neural stem cells in adult rodent brain could be achieved by using anti-CD15-SPIONs as the molecular probe; and this targeting imaging strategy has the advantage of a rapid in vivo monitoring of the subpopulation of endogenous NSCs in adult brains.

## 1. Introduction

Neural stem/progenitor cells (NSCs) persist in the adult rodent brain and have been identified in the subventricular zone (SVZ) of the lateral wall of the lateral ventricle and the hippocampus. The SVZ is the largest source of progenitors in the brain. Primary progenitors in this region correspond to type B cells, which have properties of astrocytes. These cells divide to generate transit-amplifying type C cells, which generate new neurons that migrate along a well-defined pathway, the rostral migratory stream (RMS), to the olfactory bulbs (OBs) where they differentiate into interneurons [1].

The SVZ retains a significant level of plasticity, where the generation and flow of cells change in response to signals from the anatomically remote areas of the brain or even from the external environment of the organism. Following cerebral ischemia [2], traumatic brain injury [3], or stroke [4], NSCs and neuroblasts respond by proliferation and migration towards damaged brain regions and subsequently differentiate into the phenotype of the destroyed cells [5, 6]. Disturbed adult neurogenesis has also been found in neurodegenerative diseases such as Huntington's disease, Alzheimer's disease, and Parkinson's disease [4, 7, 8]. The intrinsic sensitivity and responsiveness of SVZ to disease states raise the prospect of direct manipulation of endogenous NSCs to develop new cell replacement strategies to enhance the neurogenic response of the SVZ and contribution to brain repair [9, 10].

Development of methods capable of in vivo monitoring and tracking of endogenous NSCs would be of great benefit to the understanding of the plasticity of the SVZ under normal and pathological conditions and will be essential to ensure the safety and efficacy of neurogenesis-based therapies. To date, numerous studies had investigated the adult neurogenesis and NSC migration from SVZ by using the methods of thymidine [11], retroviruses [12], bromodeoxyuridine (BrdU) labeling [13], and transgenic systems [14, 15], whereas all of these labeling methods rely on ex vivo processing of tissue, thus limiting the ability for longitudinal studying of the neurogenesis and migration throughout the brain within a living animal.

It has been reported that magnetic resonance spectroscopy can be used to in vivo monitor endogenous NSCs based on a distinct peak of NSCs in the spectroscopic profile [16]. However, this metabolic biomarker, previously believed to be exclusive to NSCs, was demonstrated to be nonspecific for NSCs [17]. Recently, injection of micron-sized particles of iron oxide (MPIOs) or superparamagnetic iron oxide nanoparticles (SPIONs) into the lateral ventricle [18–22] or directly into the anterior SVZ [21] has been demonstrated successfully in situ to label endogenous NSCs in adult or postnatal developing rodent brains; as such, NSCs migration under normal condition [18–22] or migration towards hypoxia-ischemia lesion sites under pathological conditions could be monitored in vivo by MR imaging [23]. These approaches offer the unique advantage of in situ labeling and in vivo study of NSCs. However, these strategies suffer from some limitations. For example, the injected MPIOs could result in a large degree of signal inhomogeneity (loss of signal intensity) and image distortion on the MR images, which hampers the visualization of the SVZ and beginning of the RMS. This shortcoming could be overcome in part either by combined use of transfection agents such as poly-L-lysine or protamine sulphate or by reducing the number of MPIOs used [20] or decreasing the size of the iron oxide particles needed [21]. Most importantly, these labeling strategies suffered from an intrinsic limitation of nontargeting [18–23]. The iron oxide particles used were unmodified and have a low labeling efficiency (~30%) [19]. After administration, these iron oxide particles were nonselectively endocytosed by all cells residing within the SVZ but not exclusively by NSCs; therefore the migrating dynamics of cells with RMS were essential to transport iron oxides and play a critical role in MR imaging of endogenous NSCs.

Previous cell biological study showed that a cell surface biomarker, the LeX antigen, which is the trisaccharide 3-fucosyl-N-acetyllactosamine, also known as SSEA-1 (stage-specific embryonic antigen 1) or CD15 antigen, was highly and specifically expressed by adult mouse NSCs and shed into their microenvironment within the SVZ [24]. In view of this specific expression of CD15 antigen by a subpopulation of adult NSCs, in this study we investigate the feasibility of in vivo targeted MR imaging of endogenous NSCs in healthy adult rodent brains by using anti-CD15 monoclonal antibody-conjugated SPIONs (anti-CD15-SPIONs) as the molecular probe.

## 2. Materials and Methods

### 2.1. Animals

Adult 8–10-week-old C57BL/6J mice (weighing 20–25 g) were obtained from the Laboratory Animal Center of Sun Yat-Sen University. All animals were housed in a standard animal facility with 12 h on/off light conditions and allowed standard food and water ad libitum. All experimental procedures adhered to the guidelines for the care and use of laboratory animals and the ethical review process of our institution and were approved by the Institutional Animal Care and Use Committee.

### 2.2. Characterization of Anti-CD15-SPIONs

Commercially available SPIONs (Miltenyi Biotec, Bergisch Gladbach, Germany), conjugated with a monoclonal rat anti-mouse CD15 IgM antibody, stabilized with sodium citrate, and approved for clinical use in humans as magnetic cell separators, were used as the targeted probe. These iron oxide particles were composed of a biodegradable, nontoxic dextran-based ferromagnetic matrix, and their nominal overall mean diameter was approximately 50 nm. Assuming a diameter of 30 nm for the magnetic core, there were typically 10–200 antibody molecules per particle [25]. SPIONs conjugated with anti-mouse IgM antibody (Miltenyi Biotec, Bergisch Gladbach, Germany) were used as nontargeted control probe. To verify the conjugation of anti-CD15 antibody to the SPIONs, anti-CD15-SPIONs were tested with FITC-conjugated anti-mouse IgM (1 : 200, Chemicon, CA, USA) for 30 min and observed under a fluorescence microscope (TE2000-U, Nikon Company, Japan). The actual average size of anti-CD15-SPIONs used was determined by using a transmission electron (JEM-2010HR, JEOL, Japan) operated at 160 kV. Their actual iron content was measured by inductively coupled plasma optical emission spectrometry using a polarized Zeeman Atomic Absorption Spectrophotometer (AAS, Z-2000, Hitachi, Japan) and their *r*_2_ relaxivity was assessed at 1.5-T MR scanner (Philips Intera, Best, Netherlands) by measuring particles in vitro at room temperature (~25°C).

### 2.3. Cell Sorting Using Anti-CD15-SPIONs

To verify the specific expression of CD15 antigen of NSCs in SVZ, acutely isolated SVZ cell suspensions were labeled with anti-CD15-SPIONs and then were sorted using a magnetic-activated cell sorting method according to the manufacturer's instructions. In brief, single cell suspension was obtained from dissociated SVZ of five adult mouse brains, as described previously [24]. Then the cells were incubated with anti-CD15-SPIONs for 30 min on ice. After being washed twice with 0.1 M phosphate-buffered saline (PBS), cells were sorted on a CliniMACS system (CliniMACS, Miltenyi Biotec, Bergisch Gladbach, Germany). The sorted CD15 positive cells were collected and then were cultured in a plastic flask at a density of 5 × 10^4^ viable cells/cm^2^ in a humidified incubator at 37°C with 95% air and 5% CO_2_. The culture medium was serum-free DMEM/F12 (Dulbecco's modified Eagle's medium: nutrient mixture F12, 1 : 1, Gibco, NY, USA), supplemented with components which consisted of basic fibroblast growth factor (bFGF, 20 ng/mL, R&D systems, MN, USA), epidermal growth factor (EGF, 20 ng/mL, R&D systems), and B27 Supplement (Gibco). The culture medium was replenished regularly until typical neurospheres were generated. The generated neurospheres were identified and characterized by using immunofluorescence staining for Nestin and CD15. For immunofluorescent staining, cells were fixed with 4% paraformaldehyde for 45 min. Then, the cells were incubated with the primary antibodies against Nestin (1 : 200, Chemicon, CA, USA) and against CD15 (1 : 200, BD Pharmingen, CA, USA) overnight at 4°C in 0.1 M PBS. Afterwards, the cells were incubated with the secondary antibody, Cy3-conjugated anti-mouse IgG (1 : 100, Chemicon, CA, USA), or FITC-conjugated anti-mouse IgM (1 : 200, Chemicon, CA, USA) for 30 min.

To determine whether the subpopulation of sorted CD15 positive cells exhibit the stem cell properties, the obtained neurospheres were then transferred to the culture medium containing 10% fetal bovine serum (FBS, Gibco) to induce lineage differentiation. After 7–10 days of induction, cells were identified by immunofluorescence staining for neural cell phenotype specific markers including glial fibrillary acidic protein (GFAP), microtubule-associated protein 2 (MAP2), and O4. Differentiated cells were incubated with the primary antibodies against GFAP (1 : 100, Thermo Fisher Scientific, CA, USA) or MAP2 (1 : 100, Thermo Fisher Scientific, CA, USA) or O4 (1 : 100, Chemicon, CA, USA) overnight at 4°C in 0.1 M PBS; then Cy3-conjugated secondary IgG antibody (1 : 100, Chemicon), TR-conjugated secondary IgG antibody (1 : 100, Santa Cruz, CA, USA), or FITC-conjugated secondary IgM antibody (1 : 100, Chemicon) was applied to determine whether these cells could demonstrate glial differentiation, neuronal differentiation, and oligodendrocytic differentiation, respectively. Fluorescence images were obtained on a fluorescence microscope (TE2000-U, Nikon Company, Japan).

### 2.4. In Vitro Binding of Anti-CD15-SPIONs

To detect the in vitro binding of anti-CD15-SPIONs to NSCs, the obtained neurospheres were triturated until single cell suspension was achieved. 10^6^ single cells were incubated with 20 *μ*L anti-CD15-SPIONs at 4°C for 15 min in 80 *μ*L 0.1 M PBS. Then the cells were washed three times with PBS by centrifugation (5 min, 300 ×g, 25°C) to remove excess unbound particles. The presence of anti-CD15-SPIONs binding was verified by Prussian blue staining. The average iron content of cells was determined by AAS and the distribution of nanoparticles was determined by using transmission electron microscopy. The cells with anti-CD15-SPION binding were assessed by using in vitro MR imaging. The same number of untreated NSCs and cells incubated with nontargeted SPIONs or anti-CD15 mAb alone under the same condition was used as negative controls.

For Prussian blue staining, cells were fixed with 4% glutaraldehyde, washed, incubated for 30 minutes with 10% potassium ferrocyanide (Perls reagent) in 15% hydrochloric acid, and then washed and counterstained with nuclear fast red. For iron content measurement, cells were suspended in 10 mL 1 M HCl solutions for at least 24 hours. Then, the iron concentration of cells was determined by AAS. For electron microscopy, cells were fixed in 3% glutaraldehyde-cacodylate buffer at 3°C overnight and then fixed for 1 h in 1% OsO4. After being dehydrated in graded dilutions of ethanol, cells were embedded in artificial resin (Epon, Merck, Darmstadt, Germany) and processed for electron microscopy. Thin sections of the cell probes were evaluated unstained (i.e., without double staining with uranyl acetate and lead citrate) to prevent false-positive findings. The presence and localization of SPIONs were observed under transmission electron microscopy (CM-10, Philips, Eindhoven, Netherlands) at 60–80 kV. For in vitro MR imaging, the cell pellets were collected in 1.5 mL EP tubes and resuspended in 50 *μ*L 4% gelatin solution and then imaged.

### 2.5. In Vitro MR Imaging

In vitro MR imaging was performed at a clinical 1.5-T MR scanner (Intera, Philips Medical Systems, Best, Netherlands) and a circular 11 cm surface coil was used. MR pulse sequences included two-dimensional T2-weighted fast spin-echo sequences (repetition time/echo time msec, 2600/100; turbo spin-echo factor, 15; matrix, 384 × 256; number of signals acquired, 2; section thickness, 1.5 mm; field of view, 90 mm) and two-dimensional T2^*∗*^-weighted fast field echo sequences (210/18; matrix, 304 × 256; flip angel, 30°; number of signals acquired, 2; section thickness, 1.5 mm; field of view, 90 mm). T2 relaxation data were acquired by using a multi-spin-echo sequence. The following parameters were used: repetition time, 2000 ms; stepped echo time, 20–160 msec for eight steps; echo spacing, 20 msec; field of view, 90 mm; matrix, 256 × 256; section thickness, 1.5 mm; number of signals acquired, 2. T2 maps were calculated from T2 relaxation data with the available software tools provided by the manufacturer, which are based on least-squares algorithms [26]. On T2 maps, T2 values were derived by means of region-of-interest measurements. During measurement, a circular region of interest was used with a minimum of 15 pixels per region. Each experiment of in vitro MR imaging was repeated eight times. For measurement of *r*_2_ relaxivity of the nanoparticles, T2 relaxivity (s^−1 ^mM^−1^) was calculated through the curve fitting of 1/T2 relaxation time (s^−1^) versus the magnetic atoms (Fe) concentration (mM).

### 2.6. Surgical Injection

Thirty-six 8–10-week-old adult C57BL/6J mice were randomly divided into three groups: targeted group (*n* = 12), in which animals received stereotactic injection of 7 *μ*L anti-CD15-SPIONs; nontargeted group (*n* = 12), in which 7 *μ*L nontargeted IgM SPIONs were injected; medium alone group (*n* = 12), in which 7 *μ*L anti-CD15 mAb alone (*n* = 9) or 7 *μ*L PBS (*n* = 3) was injected. Before injection, the iron content of the nontargeted SPIONs was adjusted to be the same as that of the anti-CD15-SPIONs by dilution with PBS. For injection, mice were anesthetized using isoflurane and were placed in a stereotaxic frame. The head was shaved and ~1 cm incision was made to expose the skull. The injection into the anterior portion of the right lateral ventricles (stereotaxic coordinates: 0.95 mm lateral to bregma, 0.02 mm rostral to bregma, and 2.6 mm deep from the pial surface) was performed by an author (X.M.Z, with 3-year experience with microsurgical procedures), using a 26s gauge needle attached to a 10 *μ*L Hamilton syringe mounted on a microinjector. The medium was slowly injected at a constant rate of 0.5 *μ*L/min. After injection, the needle was left in place for additional 5 min and then slowly withdrawn. The skin over the skull was sutured closed and animals were placed in separate, heated cages and monitored until fully recovered. Brain MR imaging was performed before injection (baseline data) and at 1, 3, 7, and 10 days after injection.

### 2.7. In Vivo MR Imaging

After intraventricular injection, brain MR imaging was performed at 7.0T micro-MR scanner (PharmaScan, Bruker, Germany) with a 23 mm mouse brain coil. During MR imaging, mice were positioned in a plastic holder with a stereotaxic head-frame and anesthetized by isoflurane (1–1.5% at 0.8–1.0 L/min air flow via a nose cone) with respiratory monitoring. Axial, coronal, and sagittal brain images were obtained. The pulse sequences included two-dimensional T2^*∗*^-weighted fast low angle shot gradient echo sequence (400/3.5; flip angle, 30°; matrix, 256 × 256; field of view, 30 mm; number of signals acquired, 8; section thickness, 0.5 mm; section gap, 0) and two-dimensional T2-weighted turbo rapid acquisition with relaxation enhancement sequence (6000/60; matrix, 256 × 256; field of view, 30 mm; number of signals acquired, 6; section thickness, 0.5 mm; section gap, 0). On T2^*∗*^-weighted imaging, the signal intensity of RMS was measured by an author (J.S., with more than 12-year experience with MR imaging), using the technique of region of interest with a minimum size of 50 pixels and the decrease of signal intensity was normalized to the contralateral normal brain parenchyma.

### 2.8. Histology

In animals that received anti-CD15-SPION or IgM SPION injection, two animals of each of them were randomly sacrificed for histological assessment after MR imaging at 1 day and 3 days after injection. In animals that received anti-CD15 mAb or PBS injection, one animal was randomly sacrificed at 1 day and 3 days after injection. The animals were anesthetized with a dose of 10% chloral hydrate (350 mg/kg) intraperitoneally and were then transcardially perfused with saline followed by 4% paraformaldehyde in PBS. The brains were removed, fixed in 10% paraformaldehyde overnight, and then cryoprotected in 30% sucrose solution. Contiguous 5 *μ*m thickness sagittal sections were cut and processed for diaminobenzide- (DAB-) enhanced Prussian blue staining to determine the distribution of nanoparticles and immunofluorescence staining for CD15 and Nestin to verify the presence of anti-CD15 SPIONs.

For DAB-enhanced Prussian blue staining, sections were incubated with 2 mL Prussian blue solution containing 15% hydrochloride and 10% potassium ferrocyanide (II) trihydrate for 30 min at 37°C and then reacted with unactivated and activated (containing 0.03% hydrogen peroxide) 0.014% diaminobenzide for 15 minutes each, washed three times, and then counterstained with nuclear fast red. For immunofluorescence staining, sections were incubated with the primary antibodies against Nestin (1 : 200, Chemicon) or CD15 (1 : 200, BD Pharmingen) overnight at 4°C. The secondary antibody Cy3-conjugated anti-mouse IgG (Chemicon) or FITC-conjugated anti-mouse IgM (Chemicon) was applied for 30 min at room temperature. DAPI (1 : 1,000, Sigma–Aldrich, St. Louis, MO) was used to label the nuclei. Fluorescence images were obtained on a confocal microscope (LSM 510, Carl Zeiss Inc., Germany) or a fluorescence microscope (TE2000-U, Nikon Company, Japan).

### 2.9. Statistical Analysis

Data were expressed as mean ± SD. Analysis of variance significance (ANOVA) was performed to test for statistical significance in T2 values between cells incubated with anti-CD15-SPIONs and control cells. The normalized signal intensity of RMS was compared among animals injected with different medium by using a repeated-measures one-way ANOVA test, followed by the Bonferroni post hoc test for multiple pairwise comparisons among different times. Statistical analysis was performed with SPSS 13.0 software for Windows. *P* < 0.05 was considered to indicate a statistically significant difference.

## 3. Results

### 3.1. Characterization of Anti-CD15-SPIONs

**Figure 1:**
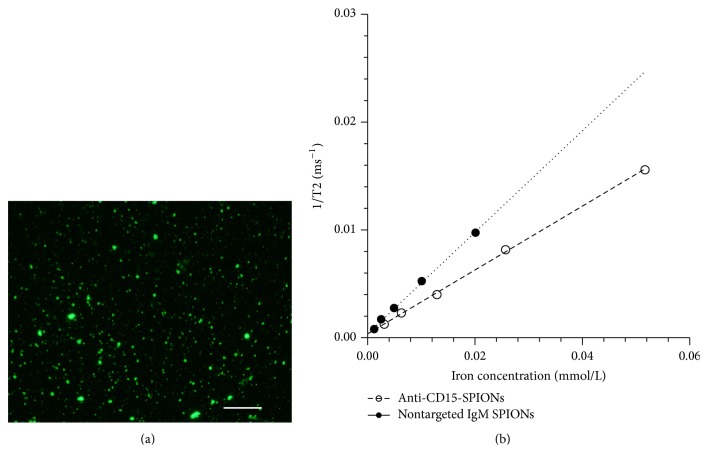
Characteristics of anti-CD15-SPIONs. Fluorescence micrograph (a) shows that anti-CD15-SPIONs were substantially positive for anti-mouse IgM staining (bar = 100 *μ*m). Graphs (b) show *r*_2_ relaxivity of anti-CD15-SPIONs and nontargeted IgM SPIONs (b).

The obtained anti-CD15-SPIONs were strongly positive for anti-CD15 fluorescence staining. Their actual average size was 73.77 ± 10.11 nm, as determined by transmission electron microscopy, and the iron concentration was 2.58 *μ*mol/mL. The measured *r*_2_ of anti-CD15-SPIONs and nontargeted IgM SPIONs was 0.30 × 10^6^ and 0.468 × 10^6^ mol^−1^·s^−1^, respectively (Figure 1).

### 3.2. Cell Sorting

**Figure 2:**
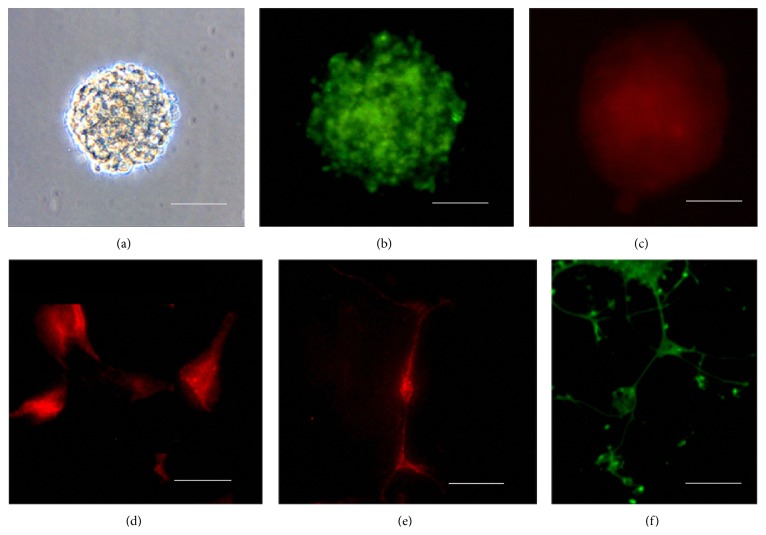
Stem cell properties of CD15 positive cells sorted from SVZ. Light microscopy micrograph shows that the sorted CD15 positive cells from SVZ could generate typical neurospheres after in vitro culturing (a). Fluorescence micrographs show that these neurospheres were positive for CD15 staining (b) and weakly positive for Nestin staining (c) and could be differentiated into cells, which are positive for GFAP (d) or MAP2 (e) or O4 staining (f). Bars in (a), (b), (c), and (f) equal 50 *μ*m; bars in (d) and (e) equal 25 *μ*m.

After being sorted by anti-CD15-SPIONs, the obtained cell subpopulation from mouse SVZ could self-renew and generate numerous neurospheres after 72 h of culturing (Figure 2(a)). Immunofluorescence staining showed that these neurospheres were positive for anti-CD15 staining but weakly positive for anti-Nestin staining (Figures 2(b) and 2(c)). After induction, the differentiated cells were positive for GFAP or MAP2 or O4 staining (Figures 2(d), 2(e), and 2(f)).

### 3.3. In Vitro Cell Binding of CD15-SPIONs

**Figure 3:**
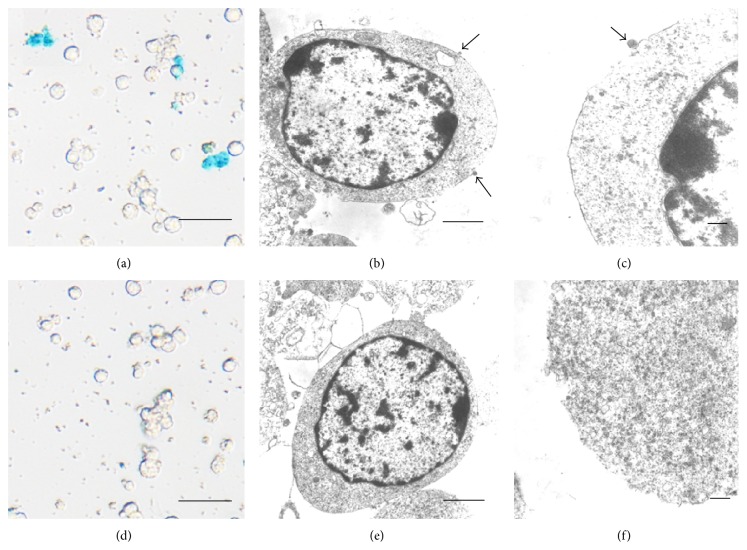
Histologic assessment of in vitro cell binding of anti-CD15-SPIONs. Prussian blue staining (a, d) and electron microscopy micrographs (b, c, e, f) demonstrate that the positive SPIONs (arrows) are bound to the cell membrane after cells were incubated with anti-CD15-SPIONs (a, b, c), while there is absence of positive SPIONs when cells were incubated with nontargeted SPIONs (d, e, f). Bars in (a) and (d) equal 25 *μ*m; bars in (b), (c), (e), and (f) equal 1 *μ*m.

After incubation with anti-CD15-SPIONs, cells showed positive staining for CD15. Unlike endosome incorporation of iron oxide nanoparticles present in exogenous stem cell labeling, the anti-CD15 SPIONs were bound to the cell membrane via antigen-antibody interaction as revealed by Prussian blue staining and electron microscopy (Figure 3). No high-density electron particles of the iron oxide nanoparticle were present within the cytoplasm.

**Figure 4:**
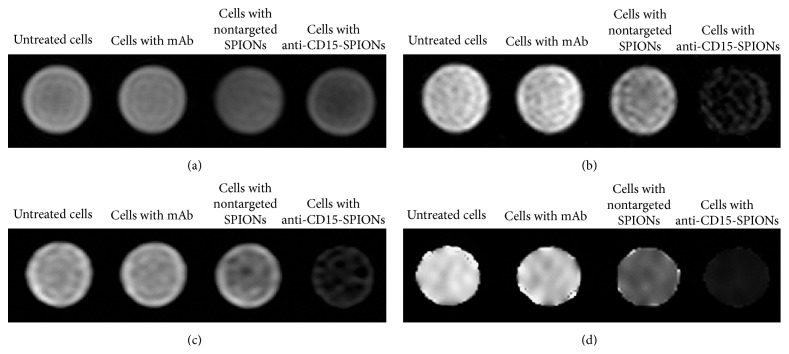
In vitro MRI of cells binding to anti-CD15-SPIONs. Cells incubated with anti-CD15 SPIONs show decreased signal intensity on T1-weighted imaging (a), T2-weighted imaging (b), and T2^*∗*^-weighted imaging (c) and T2 map (d) in comparison with negative control cells.

**Figure 5:**
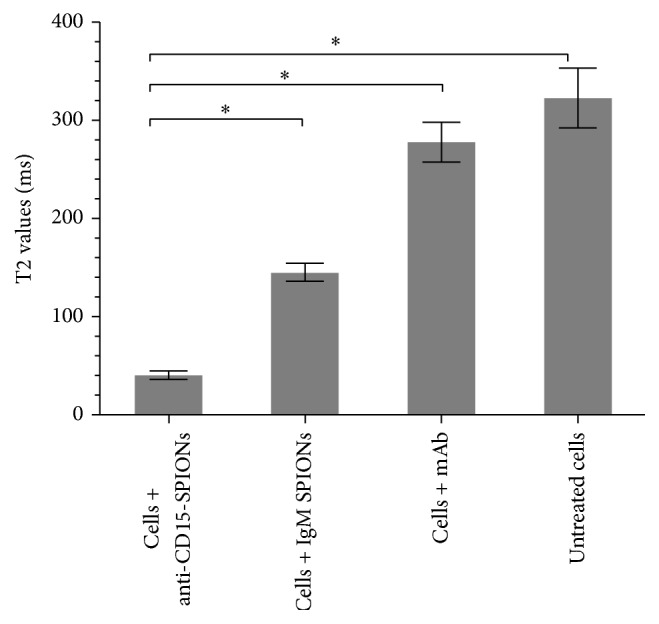
Graphs show T2 values of cell binding. T2 values of cells incubated with anti-CD15-SPIONs were lower than control cells. ^*∗*^ *P* < 0.05.

Compared with control cells, cells incubated with anti-CD15-SPIONs showed significantly decreased signal intensity on T2-weighted imaging and T2^*∗*^-weighted imaging (Figure 4). T2 values of cells incubated with anti-CD15-SPIONs, nontargeted SPIONs, anti-CD15 mAb, and untreated cells were 40 ± 4 ms, 145 ± 9 ms, 278 ± 20 ms, and 323 ± 30 ms, respectively (Figure 5). T2 value of cells labeled with anti-CD15-SPIONs was eight times shorter than that of unlabeled cells (*P* < 0.001). The mean iron concentration of cells incubated with anti-CD15 SPIONs was 19.279 pg in a single cell.

### 3.4. In Vivo MR Imaging

**Figure 6:**
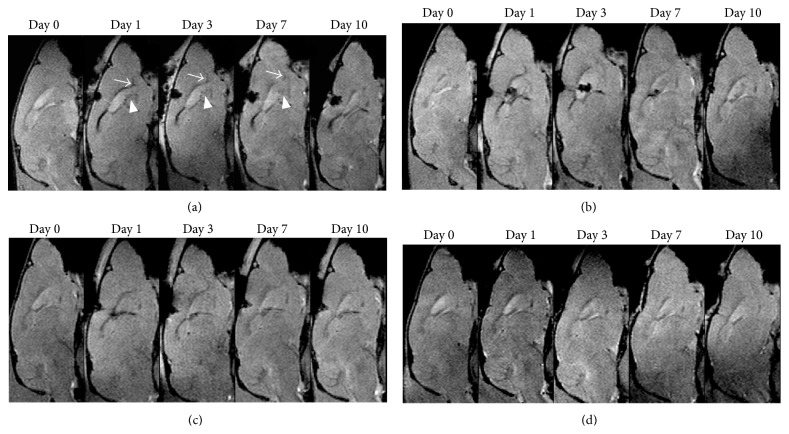
In vivo MR imaging of endogenous NSCs. Serial sagittal T2^*∗*^-weighted images showed that linear (arrows) and spotty hypointense signal (arrowheads) appear in the beginning of RMS and SVZ and in animals injected with anti-CD15-SPIONs (a); these hypointense signal patterns persist to 3 days after injection and are almost invisible by 7 days and disappear by 10 days after injection. No such developing signal intensity is found in the SVZ and RMS in animals injected with nontargeted SPIONs (b) or mAb (c) or PBS (d) alone.

Baseline data showed that RMS was visualized as a linear structure with slight hypointense signal on T2-weighted imaging and T2^*∗*^-weighted imaging compared with surrounding cerebellar parenchyma. At 1 day after intraventricular injection of anti-CD15-SPIONs, spotty hypointense signal was present in SVZ and linear hypointense signal in the beginning of the RMS on T2-weighted imaging and T2^*∗*^-weighted imaging. This decreased signal intensity persisted to 3 days after injection and returned to almost baseline level by 7 days after injection while it almost disappeared by 10 days after injection. Note that there was no hypointense artifact associated with the iron oxide particles found within and around lateral ventricles. In contrast, there was no such developing hypointense signal found in the SVZ and RMS of animals injected with either nontargeted SPIONs or antibody or PBS alone during the entire study period (Figure 6).

**Figure 7:**
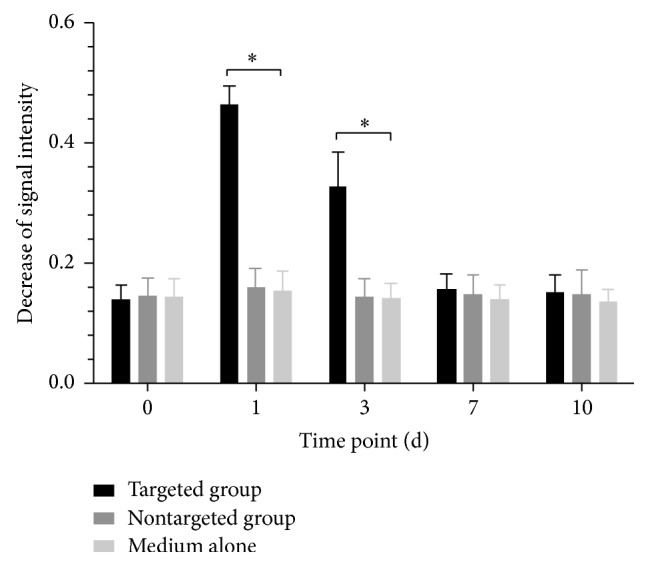
Graphs show the time of course of T2^*∗*^ signal intensity change of RMS. ^*∗*^ *P* < 0.05 compared to controls.

The measured T2^*∗*^ signal intensity of RMS of each group was shown in Table 1. At 1 day and 3 days after injection, the signal intensity was significantly lower in animals receiving anti-CD15-SPIONs (*P* < 0.01) but recovered over time, to preinjection level until 7 days after injection (*P* < 0.05). No statistical difference was found between animals injected with nontargeted SPIONs and those with simple mAb or PBS at each time point (*P* = 0.172–1.000) (Figure 7).

**Figure 8:**
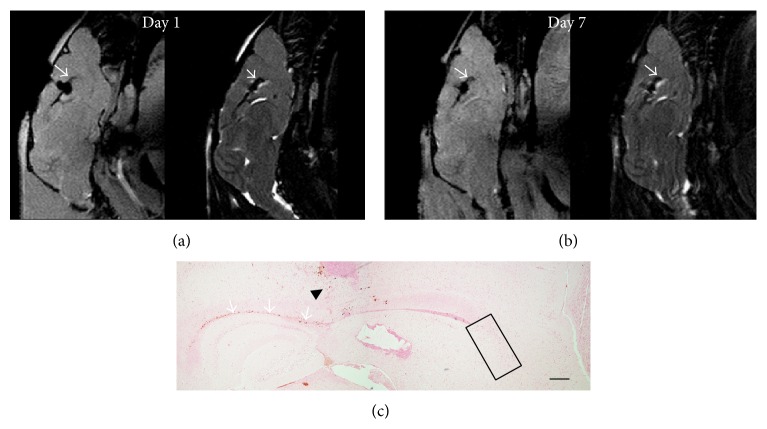
Anti-CD15-SPIONs injected into the corpus callosum. After injection of anti-CD15-SPIONs into the corpus callosum, the corpus callosum (arrows) shows decreased signal intensity on T2^*∗*^-weighted (a) and T2-weighed images (b), which was retained until 7 days after injection. No decreased signal intensity appears in the RMS or SVZ. Prussian blue staining reveals that there were positive SPIONs in the needle track (black arrowhead) and the corpus callosum (arrows), while no positive SPIONs are present in the SVZ and RMS (black rectangle). Bar = 200 *μ*m.

Whether animals received anti-CD15 or nontargeted SPIONs, a hypointense signal was present in the corpus callosum. To determine whether the decreased signal observed in the RMS resulted from the rostral diffusion of nanoparticles along the corpus callosum, 7 *μ*L anti-CD15-SPIONs were dedicatedly injected into the corpus callosum in another three animals. After injection, the expected hypointense signal occurred in the corpus callosum, whereas no concurrent hypointense signal intensity was found in the RMS even though the decreased signal intensity of the corpus callosum persisted to 7 days following injection. DAB-enhanced Prussian blue staining confirmed that there were no anti-CD15-SPIONs present within the SVZ and the RMS (Figure 8).

### 3.5. Histology

**Figure 9:**
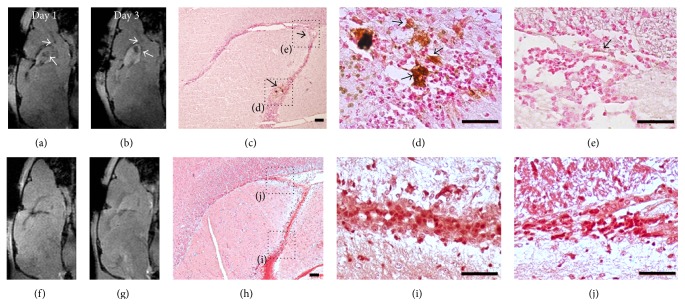
Histologic assessment of the distribution of SPIONs. At 1 day and 3 days after injection, T2^*∗*^-weighed images show hypointense signal in the SVZ and the beginning of RMS (arrows) in an animal injected with anti-CD15-SPIONs ((a), (b)). The corresponding DAB-enhanced Prussian blue staining reveals that anti-CD15-SPIONs are localized around cells and extracellular matrix (arrows) within SVZ ((c), (d)) and RMS (e) at 3 days after injection, which is well matched with MRI findings. In an animal that received nontargeted SPION injection, no hypointense signal is present in the SVZ and RMS ((f), (g)). DAB-enhanced Prussian blue staining reveals that no iron oxide nanoparticles were found in the SVZ or RMS. Bars in (c) and (h) equal 100 *μ*m; bars in (d), (e), (i), and (j) equal 50 *μ*m.

DAB-enhanced Prussian blue staining revealed that there were positive cells within SVZ and RMS of animals injected with anti-CD15-SPIONs at 1 day and 3 days after injection. A majority of nanoparticles were present not only in cells but also within extracellular matrix (Figure 9). The location of these SPIONs was well matched with hypointense signal observed on MR imaging. Immunofluorescence staining confirmed the presence and distribution of anti-CD15-SPIONs in the SVZ and RMS. Confocal microscopy images showed that those cells binding anti-CD15-SPIONs were positive for CD15 staining, whereas they were negative for Nestin staining. In contrast, no positive SPIONs were found in the SVZ or RMS of animals injected with nontargeted SPIONs or mAb or PBS alone (Figure 9).

## 4. Discussion

Our study results demonstrated that anti-CD15 antibody-conjugated SPIONs delivered by intraventricular injection could specifically accumulate around a subpopulation of NSCs with a CD15 phenotype in the SVZ and RMS of adult rodent brains. Upon this targeted binding, the in vivo distribution and location of NSCs could be readily visualized on MR imaging. The targeted imaging of endogenous NSCs could be achieved rapidly 1 day after delivery and longitudinally persists for at least 1 week in the same living animals.

SVZ is a continual germinal zone surrounding the ventricles, which expands prominently during the latter third trimester of prenatal period and remains constant thereafter throughout the mammalian life. It is the largest source of NSCs and transit-amplifying progenitor cells that can generate progeny during mammalian forebrain development [27, 28]. Enormous progress has been made in recent decades in uncovering the contributions of NSCs in SVZ to normal brain development. It is becoming increasingly imperative to investigate a tool with the ability to identify and track NSCs in vivo, which could be greatly helpful for the follow-up of deviating NSC migration patterns and designing therapeutic interventions for exploration of their curative potential.

However, endogenous NSCs are a rare cell population in adult mammalian brains, and little is known about their unique biological characteristics. Genes expressed by adult neural stem cells include Nestin, Musashi, Notch1, and GFAP, but other neural cell types also express these. Moreover, these markers are intracellular. The nonspecific expression and intracellular location limited their usefulness for stem cell positive identification. A more generally useful marker would be surface molecule allowing stem cell localization and clarification from other neural cells. Recently, LeX/SSEA-1/CD15 was indicated as a specific gene product that is exclusively expressed on the surface of distinct pools of adult NSCs and is shed in the microenvironment [23]. This characterization of LeX expression by adult CNS stem cells aids in vivo identification of these important cells. In this study, SVZ cells sorted by anti-CD15-SPIONs demonstrated the ability to self-renew and generate neurospheres and could be differentiated to three different neural cell types. This further corroborates their potential role of a useful cell type marker for in vivo identification of adult NSCs.

Previous animal studies demonstrated that direct injection of MPIOs or SPIONs with canonic reagents into the lateral ventricles could be used to label NSCs within SVZ. The iron oxide particles were endocytosed by cells within SVZ and then could be distinguished from the surrounding tissue as they appear as hypointense spots on T2^*∗*^-weighted MR images. By virtue of the migrating nature of NSCs along the RMS to the olfactory bulb, the NSC dynamics could be followed in vivo by MR imaging [18–23]. However, it was shown that the uptake of MPIOs or iron oxide nanoparticles by the precursors in the SVZ is small (~30%) and 4% iron oxide particles were likely engulfed by ependymal cells [19]. There is also a possibility that MPIOs could travel along the RMS and then be endocytosed locally by other cells. Moreover, a varied, prolonged period of time was required for in vivo tracking NSCs by using MPIO or cationic protein-complexed SPIONS, for example, 2 weeks to 5 weeks were needed for tracking NSCs in adult mature brain [19–22] and 1 week to 2 weeks were required for tracking NSCs in neonatal brain [23]. On the other hand, the intracellular and nonspecific retention of iron oxide particles within cells could persist for a long period of time, even until 8 weeks after injection [18]. Such prolonged retaining of MPIOs or SPIONs could probably cause detrimental effects on the biological behavior of labeled cells, since MPIOs while being inert inside a cell could have significant impact on cellular processes due to their large particle size and styrene/divinylbenzene coating [29].

In our study, on the basis of specific expression of CD15 antigen on the surface of distinct pools of NSCs in adult mice, a targeting SPION with anti-CD15 mAb as the ligand was used for in vivo MR imaging of the NSCs. After intraventricular delivery of anti-CD15-SPIONs, the endogenous NSCs, those residing in the SVZ and RMS in adult mouse brain, were detected as hypointense signal at a rapid time, that is, 1 day after injection. Furthermore, the presence of iron oxide nanoparticle persisted in a relatively short period of time (7 days after injection). Histology confirmed that these nanoparticles are targeted to cell surface of CD15 positive NSCs and their extracellular environments. Together with previous observation of histologic distribution of CD15 antigen in SVZ [24], our findings suggested that targeted MR imaging of endogenous NSCs in adult mouse brain was successfully achieved by using CD15-conjugated SPIONs.

Unlike MPIO or SPIO complex, the use of CD15-targeted SPIONs has several advantages. Since the small size of the nanoparticles and a small volume are needed, the image of entire SVZ and RMS in our study was almost free from the susceptibility artifact. Due to the specific binding between antibody and CD15 antigen, the targeting nanoparticles were only present around CD15 positive NSCs and their surrounding matrix; the resulting signal change on MR imaging is high likely to reflect the actual change in NSCs plasticity. It has been revealed that in mice many new neurons reach the OB by two days and a majority arrives in the OB by day 6 [30]. Therefore, tracking NSC migrating patterns in such highly active state would greatly benefit from our strategy. Moreover, in comparison with internalization of larger MPIOs or SPION complex, cellular surface binding of the biodegradable nanoparticles used in our study would be more favorable for in vivo cellular imaging as the intact biological behavior of cells could be less likely affected.

A number of limitations exist in the current study. First, like targeting immunotherapy and targeting imaging where an antibody ligand is coupled to targeting probe, a common problem of using CD15-targeted nanoparticles is that introduction of heterologous antibody could result in host immunological reaction. In our study, a homologous murine monoclonal antibody was conjugated to iron oxide nanoparticles with which the immunological reaction might be alleviated. With the advancement of molecular biology, use of specific single-chain Fv antibody would be potentially preferred in the future owing to the advantages such as decreased immunogenicity and favorable pharmacokinetics/biodistribution profiles [31]. The second is the lack of the immunohistological staining for several other neural cell phenotypes, such as ependymal cells, neuroblasts, oligodendrocytes, and microglia, and the absence of quantitative analysis of labeled cells. Previous study had characterized CD15 positive population in the SVZ [24]. The results had shown that there was little overlap between CD15 phenotype and other phenotypes such as GFAP, *β*-tubulin III, mCD24, and PSA-NCAM; and 4% of acutely isolated SVZ cells are CD15 positive. Third, our investigation was only performed in normal mouse brain. Future studies are needed to address the in vivo MR imaging of NSC migration in adult diseased brains.

In summary, our results demonstrated that in vivo targeted MR imaging of endogenous NSCs in adult mouse brains could be successfully achieved by intraventricular delivery of anti-CD15 mAb-conjugated SPIONs. Use of these nanoparticles as the molecular probe could allow a rapid in vivo tracking of adult NSCs with MR imaging. Such in vivo targeted MR imaging of the NSCs can be used to reflect the neurogenesis size of the SVZ in the healthy brain and potentially the time course of activation of the NSCs within the SVZ in diseased brain, resulting in improved understanding of neurogenesis in normal mammalian brains and its alterations during diseases. This direct observation of NSCs has potential as an additional diagnostic and prognostic criterion in brain diseases, as the neurogenesis in the SVZ might indicate the recovery phase and the stage and grade of the disease.

## Figures and Tables

**Table 1:** The signal intensity decrease of the RMS.

Follow-up	Targeted group	Nontargeted group	Medium alone
	(*n* = 8)	(*n* = 8)	(*n* = 8)
0 d	0.140 ± 0.024	0.156 ± 0.029	0.134 ± 0.030
1 d	0.463 ± 0.032	0.160 ± 0.031	0.155 ± 0.032
3 d	0.328 ± 0.057	0.145 ± 0.029	0.122 ± 0.025
7 d	0.237 ± 0.025	0.109 ± 0.031	0.140 ± 0.024
10 d	0.182 ± 0.028	0.118 ± 0.041	0.126 ± 0.021

*Note*. There were 8 animals yielding complete data in the animals injected with anti-CD15-SPIONs or nontargeted SPIONs or medium alone. The signal intensity decrease of the RMS was normalized to the contralateral normal brain parenchyma.
